# Unraveling the pathogenesis of *ARX* polyalanine tract variants using a clinical and molecular interfacing approach

**DOI:** 10.1002/mgg3.133

**Published:** 2015-02-25

**Authors:** Isabel Marques, Maria João Sá, Gabriela Soares, Maria do Céu Mota, Carla Pinheiro, Lisa Aguiar, Marta Amado, Christina Soares, Angelina Calado, Patrícia Dias, Ana Berta Sousa, Ana Maria Fortuna, Rosário Santos, Katherine B Howell, Monique M Ryan, Richard J Leventer, Rani Sachdev, Rachael Catford, Kathryn Friend, Tessa R Mattiske, Cheryl Shoubridge, Paula Jorge

**Affiliations:** 1 Unidade de Genética Molecular, Centro de Genética Médica Doutor Jacinto Magalhães, Centro Hospitalar do Porto, EPEPorto, Portugal; 2 Unidade de Genética Médica, Centro de Genética Médica Doutor Jacinto Magalhães, Centro Hospitalar do Porto, EPEPorto, Portugal; 3 Unit for Multidisciplinary Research in Biomedicine, UMIB, ICBAS-UPPorto, Portugal; 4 Department of Pediatrics, Centro Hospitalar do Porto, EPEPorto, Portugal; 5 Department of Pediatrics, Hospital Santa Maria Maior, EPEBarcelos, Portugal; 6 Department of Pediatrics, Hospital Distrital de Santarém, EPESantarém, Portugal; 7 Department of Pediatrics, Unidade Hospitalar de Portimão, Centro Hospitalar do AlgarvePortimão, Portugal; 8 Department of Genetics, Hospital de Santa MariaLisboa, Portugal; 9 Department of Neurology, Royal Children's HospitalMelbourne, Victoria, Australia; 10 Murdoch Childrens Research InstituteMelbourne, Victoria, Australia, 3052; 11 University of Melbourne Department of PaediatricsMelbourne, Victoria, Australia, 3052; 12 Department of Medical Genetics, Sydney Children's HospitalHigh St., Randwick, New South Wales, 2031, Australia; 13 SA Pathology at the Women's and Children's HospitalNorth Adelaide, South Australia, Australia; 14 Department of Paediatrics, University of AdelaideAdelaide, South Australia, 5006, Australia; 15 Robinson Research Institute, University of AdelaideAdelaide, South Australia, 5006, Australia

**Keywords:** Aristaless-related homeobox gene, ARX, expanded polyalanine tract, intellectual disability, pathogenic variant

## Abstract

The Aristaless-related homeobox (*ARX*) gene is implicated in intellectual disability with the most frequent pathogenic mutations leading to expansions of the first two polyalanine tracts. Here, we describe analysis of the *ARX* gene outlining the approaches in the Australian and Portuguese setting, using an integrated clinical and molecular strategy. We report variants in the *ARX* gene detected in 19 patients belonging to 17 families. Seven pathogenic variants, being expansion mutations in both polyalanine tract 1 and tract 2, were identifyed, including a novel mutation in polyalanine tract 1 that expands the first tract to 20 alanines. This precise number of alanines is sufficient to cause pathogenicity when expanded in polyalanine tract 2. Five cases presented a probably non-pathogenic variant, including the novel HGVS: c.441_455del, classified as unlikely disease causing, consistent with reports that suggest that in frame deletions in polyalanine stretches of *ARX* rarely cause intellectual disability. In addition, we identified five cases with a variant of unclear pathogenic significance. Owing to the inconsistent *ARX* variants description, publications were reviewed and *ARX* variant classifications were standardized and detailed unambiguously according to recommendations of the Human Genome Variation Society. In the absence of a pathognomonic clinical feature, we propose that molecular analysis of the *ARX* gene should be included in routine diagnostic practice in individuals with either nonsyndromic or syndromic intellectual disability. A definitive diagnosis of *ARX*-related disorders is crucial for an adequate clinical follow-up and accurate genetic counseling of at-risk family members.

## Introduction

Since its discovery in 2002, Aristaless-related homeobox gene (*ARX*; MIM# 300382; GenBank: NM_139058.2) has been implicated in X-linked intellectual disability (XLID) (Bienvenu et al. 2002; Shoubridge et al. 2010). The *ARX* gene, cytogenetically located on Xp21.3, encompasses 12.5 kb of genomic DNA and includes five coding exons encoding a 562 amino acid protein. This gene is a paired-type homeodomain transcription factor expressed predominately in the fetal and adult brain, testis, skeletal muscle, and pancreas. ARX has a critical role in brain development, particularly in GABAergic interneuron migration during cortical development (Miura et al. 1997; Kitamura et al. 2002; Stromme et al. 2002a). Hence, it is not surprising that epilepsy and structural brain malformations including lissencephaly and agenesis of corpus callosum are frequently observed in patients with *ARX* pathogenic mutations. Although patients may present with intellectual disability (ID) without additional clinical features (nonsyndromic ID), the concomitant observation of neurological deficits, with or without brain and/or genital anomalies suggests a number of recognizable syndromes. The ARX-associated spectrum of disorders includes Partington syndrome (OMIM 309510) (Partington et al. 2004), Early Infantile Epileptic Encephalopathy (OMIM 308350) (Kato et al. 2007), Agenesis of Corpus Callosum with Abnormal Genitalia (OMIM 300004) (Proud et al. 1992), and X-Linked Lissencephaly with Ambiguous Genitalia (OMIM 300215) (Kitamura et al. 2002). A genotype–phenotype relationship exists and has recently been discussed (Shoubridge et al. 2010). Despite a broad range of phenotypes associated with mutations in *ARX*, ID is a consistent clinical feature.

Although disease-causing variants occur across all five coding exons, most are located in the largest exon, exon 2. These include expansion of the first or second polyalanine tracts. In particular, duplication of 24 base pairs (bp), HGVS: c.441_464dup (referred to as dup24) in the second polyalanine tract is by far the most common pathogenic mutation in this gene. As most mutations are in this largest exon *ARX* mutation analysis is routinely confined to screening exon 2.

Report of *ARX* mutations in the literature is hampered by use of nonstandard nomenclature. Occasionally, publications describing “novel” sequence variants actually represent nomenclature errors, which are a cause of uncertainty and misinterpretation especially in medical diagnostic settings. For example, as many as seven descriptions can be identified for a single *ARX* gene variant expanding the first polyalanine tract (Stromme et al. 2002a; Wohlrab et al. 2005; Wallerstein et al. 2008; Kitamura et al. 2009; Fullston et al. 2011; Mirzaa et al. 2013). There is an obvious need for an effective molecular testing approach for *ARX* screening of ID patients and an unambiguous nomenclature for description of *ARX* variants. In this report, we have undertaken molecular analysis of the *ARX* gene using Portuguese and Australian cohorts of patients with syndromic and nonsyndromic ID. The continued characterization of *ARX* variants not only contributes to the identification of specific phenotypic features but also assists in unraveling the pathogenicity of additional rare variants. This type of information is required for accurate genetic counseling of *ARX* at-risk family members.

## Materials and Methods

### Patient recruitment

Clinical data and blood samples were collected after informed consent from the patients or their legal guardians. The National Committee for Data Protection authorized laboratory records. Relevant human research ethics committees have approved these studies. A total of 138 patients from two cohorts were enrolled for this study. Cohort 1 consisted of 101 Australian patients. Ninety patients were directly referred for polyalanine tract 1 (pA1) and tract 2 (pA2) *ARX* size-variant analysis. Thirteen patients were referred for sequencing of the entire open reading frame of *ARX* gene, including two cases that had already been tested negative for size variants in pA1 and pA2, but were included to rule out mutations elsewhere in the gene. Cohort 2 consisted of thirty-seven Portuguese patients. Twenty-four patients were referred for pA1 and pA2 *ARX* size-variant analysis. The remaining 13 patients were pulled out from a previous study consisting of the simultaneous analyses of fragile-X (*FMR1*), FRAXE (*AFF2*), and *ARX* (henceforward named multiplex screening) (Jorge et al. 2013). The selection of all subjects was based on clinical criteria assessed by the patients' geneticist or neurologist. Seventeen patients were subsequently sequenced for the entire open reading frame of *ARX* gene, based on clinical criteria (e.g. brain malformation) assessed by patient physicians. Consanguinity was excluded in the parents unless otherwise stated.

### Molecular analysis

Cohort 1 – A PCR-based approach was used to screen for expansions in pA1 and pA2 in exon 2 of *ARX*. Any changes in migration of the amplicons detected by gel electrophoresis were subsequently analyzed by bidirectional cycle sequencing reactions. The PCR and electrophoresis conditions have been described in detail previously (Tan et al. 2013). Sequencing reactions were performed using ABI Big Dye Terminator chemistry version 3.1 and purified products subjected to an automated capillary sequencing on ABI 3100 sequencer (Applied Biosystems, Foster City, CA, USA) and analysis was aided by Seqscape V2.5 (Applied Biosystems). In 13 patients the entire open reading frame was screened. Each of the five exons of *ARX* was amplified by PCR using primers designed to amplify coding and flanking noncoding sequence (Table S1). The exception to this is exon 2, for which four overlapping amplicons were used to achieve robust amplification of GC-rich regions coding for three polyalanine tracts (Tan et al. 2013). Sequence of each amplicon was subsequently confirmed as outlined above.

Cohort 2 – A multiplex-PCR method designed to amplify a portion of the second exon of *ARX* gene that includes the first two of the three tracts of repetitive alanine coding triplets was previously published by the authors (Jorge et al. 2013). Further size estimation on capillary electrophoresis allows identification of size variants: a 380 bp amplicon matches the expected normal-sized allele and a 404 bp amplicon corresponds to the dup24. Any changes in migration of the amplicons detected by gel electrophoresis were subsequently analyzed by cycle sequencing as described above for the Australian cohort. M13-tailored primers were designed to amplify all exons and intron–exon borders (Table S1) and sequencing of each of the five *ARX* exons was done following standard procedures on ABI 3130xl (Applied Biosystems, Foster City, CA, USA).

### Bioinformatics

Human Genome Variation Society (HGVS) nomenclature is stated for the *ARX* variants and refers to the GenBank mRNA entry NM_139058.2 (den Dunnen and Antonarakis 2000). The software Mutalyzer 2.0.beta-31 (www.mutalyzer.nl, HGVS nomenclature version 2.0) was used to check nomenclature of variants identified in the Portuguese and Australian cohorts. In addition, *ARX-*Locus Specific Database, LOVD (http://LOVD.nl/ARX; last accessed on March 2014) (Fokkema et al. 2011) and Human Gene Mutation Database, HGMD Professional (last accessed on April 2014) databases were crosschecked.

### Statistical analyses

A chi-square (*χ*^2^) distribution test was performed, with a significance level (*P*) of 0.05 and a confidence of 95%, to verify differences between each cohort (cohort 1 Australian and cohort 2 Portuguese). Descriptive data are presented as median with interquartile range (IQR).

## Results

### Clinical summary of patients referred for diagnostic *ARX* study

Detailed clinical description and screening outcomes for family A to Q is described in Data S1. All 138 patients enrolled for *ARX* study had ID, with 127 (92.0%) being male and 11 (8.0%) female (Table1). Forty-two patients (30.4%) were referred exclusively due to ID. Approximately half of the patients were also affected by a movement disorder or epilepsy (67/138; 48.5%). A smaller proportion of patients presented with behavior abnormalities (17/138; 12%) or with brain malformations, with and without genital malformations (12/138; 8.6%). Interestingly, the cohorts differ in regards to the presence of a movement disorder or epilepsy (*P* < 0.007) and brain malformations, with and without genital malformations (*P* < 0.001) (Cohort 2 mainly without specific *ARX*-related referral). In total, 19 patients, four of whom are female, belonging to 17 families, had an *ARX* sequence change. Sequence variants were restricted to the polyalanine coding regions in *ARX* exon 2 and positioned in pA1 (*n* = 6) and pA2 (*n* = 13) and therefore all variants alter the ARX polyalanine content. Examples of partial electropherograms of *ARX* variants identified are shown in Figure S1. Inheritance was determined in eight male patients (maternal) and one female (paternal). In one male patient, a *de novo* variant was identified (one of six for which gDNA from the parents was available; 16.7%).

**Table 1:** Clinical data of patients referred for diagnostic *ARX* study

	Cohort 1 (*N* = 101) [13 sequenced]	Cohort 2 (*N* = 37) [17 sequenced]	Total (%)
Males: *N*; Age at diagnosis (years) (median; IQR)	93; (5.5; 9.1)	34; (11; 7)	127 (92)
Females: *N*; Age at diagnosis (years) (median; IQR)	8; (3.65; 38.15)	3; (8; 7.5)	11 (8)
Family history consistent with X-linked inheritance pattern: *N*	13	3	16 (11)
Nonsyndromic ID: *N* (%)	29 (28.7)	13 (35)	42 (30.4)
Syndromic ID: *N* (%)			
With brain only or brain and genital malformations	4 (4)	8 (21)	12 (8.6)
With movement disorders and/or seizures (without information on brain or genital malformations)	56 (55.4)	11 (30)	67 (48.5)
With behavior abnormalities	12 (11.9)	5 (14)	17 (12.3)

ADHD, attention deficit hyperactivity disorder; ID, intellectual disability; IQR, interquartile range.

### Standardization of nomenclature for the mutations in the polyalanine tracts of *ARX*

Publications were reviewed and *ARX* variants descriptions were standardized and detailed unambiguously according to recommendations of the HGVS (Table2). The HGVS recommends that duplications be designated by *“dup”* after an indication of the first and last nucleotide(s) duplicated. Moreover, the recommendations state that for all descriptions the *most 3′ position* possible is arbitrarily assigned to have been changed. This does not concur with what has been previously annotated for these mutations. For example, the most common mutation, c.429_452dup(24), should be annotated as c.441_464dup according to the HGVS. Given the sequence of this region we can see that the 24 bp that are duplicated (as identified from sequencing affected patients) can either be lined up from the position c.429 or c.441 as the most *3′* position. A similar *3′* shift in nomenclature is also observed when the longer 27 bp duplication (Demos et al. 2009) and the 33 bp duplication (Reish et al. 2009) in this pA2 tract are considered (Fig. S2).

**Table 2:** Nomenclature standardization: comparison with the previously reported

Polyalanine content variation	Nomenclature proposed according to HGVS		Present study	Also described as	References
	cDNA: NM_139058.2	Protein-level: NP_620689.1			
**pA1**					
16>23	c.306GGC[17]/c.306_308[17]/c.315_335dup	p.(Ala109_Ala115dup)/p.(115Ala_7_)	dup/ins[GGC]_7_	c.304ins(GCG)_7_(GCG)_10+7_c.333ins(GCG)_7_c.333_334(GCG)_7_c.333_334ins(GCG)_7_c.333_335dup(GGC)_7_c.335ins21	Fullston et al. (2011)Stromme et al. (2002a)Stromme et al. (2002a)Wohlrab et al. (2005)Mirzaa et al. (2013)Mirzaa et al. (2013)Wallerstein et al. (2008)
16>20	c.306GGC[14]/c.306_308[14]/c.324_335dup	p.(Ala112_Ala115dup) p.(115Ala_4_)	dup/ins[GGC]_4_	Novel	
16>19	c.306GGC[13]/c.306_308[13]/c.327_335dup	p.(Ala113_Ala115dup)/p.(115A_3_)	dup/ins[GGC]_3_	c.304ins(GCG)3c.333_334(CGC)3	Stromme et al. (2002b)Stromme et al. (2002b)
16>17	c.306GGC[11]/c.306_308[11]/c.333_335dup	p.(Ala115dup)/p.(115A)	dup/ins[GGC]_1_	c.304ins(GCG)1c.333_334ins(GCG)	Fullston et al. (2011)Stromme et al. (2002b)
16<12	c.306GGC[6]/c.324_335del	p.(A112_A115del)	del12	c.321-332del	Stromme et al. (2002b)
**pA2**					
12>23 (10A-G-12A)	c.426_458dup	p.(Gly143_Ala153dup)	Not reported in this study	c.423_455dup(33 bp)	Kuwaik et al. (2012)
12>21	c.435_461dup	p.(Ala147_Ala155dup)/p.(155A_9_)	Not reported in this study	c.430_456dup(27 bp)	Stromme et al. (2003)
12>20	c.441_464dup	p.(Ala148_Ala155dup)/p.(155A_8_)	dup24	c.429_452dupc.428_451dupc.431_454dupc.464_465ins24	Bienvenu et al. (2002)Stromme et al. (2002b)Gronskov et al. (2004)
12<4	c. 441_464del	p.(Ala148_Ala155del)	del24	c.431_454delc.429del24	Wallerstein et al. (2008)Bienvenu et al. (2002)
12<7	c. 441_455del;[=]	p.(Ala151_Ala155del);[=]	del15	Novel	

HGVS: Version 2.121101.

The recurrent pA1 expansion previously described as c.304insGCG is a *duplicating insertion* and should therefore be considered a duplication, and according to the 3′ rule, must be described as c.306GGC[11] or c.315_335dup. Concerning the identified pA1 deletion, there are two alternative descriptions: c.306GGC[6] and c.324_335del. If the repeat was not sequenced but instead the size was deduced from the length of a PCR fragment or while describing a predicted (not analyzed) protein change, curved brackets were used: p.(Ala151_Ala155del).

### Clinical and molecular data of patients with an *ARX* variant

The clinical and molecular data for each of the 17 patients we are reporting with an *ARX* variant are detailed individually in Data S1 and summarized in Table3, including the age and reason for referral. Of the17 cases or variants identified, seven of these are known to be pathogenic, a detection rate of 5%. In cohort 1 there were two pathogenic mutations identified; (i) expansion of pA2 in Family G detected from the 93 patients referred and screened by *ARX* size-variation, and (ii) expansion of pA1 in Family A identified from the 13 patients referred and screened for sequencing of the entire open reading frame of *ARX* gene. In cohort 2, there were six males and one female from five families (Family H to L) identified with pathogenic expansions in pA2 from the 13 patients from the previous multiplex screening study.

**Table 3:** Summary of available clinical and molecular data of patients with an *ARX* variant

Family	Variant nomenclature	Change in ala	Age^1^	Primary referral reason/index case	Number of cases and gender	Heredity	Pathogenicity
**pA1**							
A	c.306GGC[17]/c.306_308[17]	16>23	2 months	Developmental delay and infantile seizures	1M	Maternal	Pathogenic
B	c.306GGC[14]/c.306_308[14]	16>20	2	Developmental delay, microcephaly, spasticity, and dystonia	1M	Maternal	VUS
C	c.306GGC[13]/c.306_308[13]	16>19	4	Developmental delay, intellectual, and speech delay	1M	Unknown	VUS
D	c.306GGC[10];[11]	16>17	4	Developmental delay and autistic behavior	1F	Unknown	VUS
E	c.306GGC[11]/c.306_308[11]	16>17	4	Mild developmental delay, attention deficit, and hyperactivity	1M	Unknown	
F	c.306GGC[6]/c.324_335del	16<12	10	Intellectual disability	1M	Unknown	VUS
**pA2**							
G	c.441_464dup	12>20	6 months	Developmental delay, spasms	1M	Maternal	Pathogenic
H		12>20	2; 7	Intellectual disability and congenital macrocephaly (in both)	2M^3^	Maternal	
I		12>20	4; 39	Hyperactivity and developmental delay; Dysmorphisms and dystonia of the hands	2M^4^	Maternal	
J		12>20	8	Intellectual disability, nonspecific cranio-facial dysmorphisms	1M	*De novo*	
K		12>20	5	Motor impairment, severe speech delay, and hyperactivity	1M	Maternal	
L	c.441_464dup;[=]	12>20	10	Intellectual diability and speech delay	1F (heterozygote)	Unknown	
M		12<4	4	Developmental delay	1M	Unknown	Unlikely
N	c. 441_464del	12<4	13	Learning disability	1M	Unknown	Unlikely
O		12<4	8	Learning disability, attention deficit, and hyperactivity	1M	Maternal	Unlikely
P	c.441_464del;[=]	12<4	11	Intellectual disability	1F	Unknown	Unlikely
Q	c. 441_455del;[=]	12<7	6 months	Developmental delay, nonspecific craniofacial dysmorphisms	1F	Paternal	Notpathogenic^2^

VUS, variant of uncertain/unclassified significance; F, Female; M, Male.

1 In years unless otherwise specified.

2 Polymorphism absent in 200 control samples.

3 Brothers.

4 Nephew/uncle.

### Polyalanine tract 1 variants

We identified six distinct families with a pA1 variant (Families A to F) (Table4). The pathogenic c.306GGC[17], p.(115Ala_7_) mutation in Family A leading to an increase in pA1 to 23 alanines was identified in a patient with infantile epileptic-dyskinetic encephalopathy. This variant has been reported previously and is not found in the control population (Stromme et al. 2002a; Poirier et al. 2006; Guerrini et al. 2007; Fullston et al. 2011). Table4 compares the clinical features of this case with other patients identified with this expansion. Overall, data in Table4 is compiled from 27 affected individuals and includes 8 de novo cases (Guerrini et al. 2007; Poirier et al. 2008; Shinozaki et al. 2008; Wallerstein et al. 2008; Absoud et al. 2009; Mirzaa et al. 2013), three cases of brother pairs (Guerrini et al. 2007; Cossee et al. 2011) two cases of families with maternal uncle–nephew pairs, one case of a pair of brothers and a maternal male cousin (Bruyere et al. 1999; Stromme et al. 2002a), and four cases without a family history (Mirzaa et al. 2013). There is only limited reporting of affected females in these families. The family in Cossee et al. (2011) lists a sister of the proband, with mild ID and epilepsy but no indication of age of onset or the type of seizure or movement disorder.

**Table 4:** Clinical features of patients with an *ARX* mutation leading to expansion of polyalanine tract 1 to 23 alanines

Clinical data	Previous studies (frequency) families (27 males)#	This study family A (male, 2.5 years)	Total % (frequency)
**DD/ID**	100% (27/27)	+	100% (28/28)
Age at diagnosis (median, IQR)	Onset 0–7 (median 3; range 2.25 m) (data from 9/27)	8 weeks	Onset 0–7 (median 2; range 2 m) (data from 10/28)
Formal development evaluation (IQ, median age of evaluation in years)	1/27 mild, 15/27 severe, 2/27 Profound, DD (median 3 Y)	Profound DD	1/28 mild, 15/28 severe, 3/28 Profound (median 2.5 Y)
DD onset prior to seizure onset (age at diagnosis)	22% (6/27) (median age 2 m)	+	25% (7/28)
Developmental regression with seizure onset	22% (6/27)	+	25% (7/28)
**Evaluation of MRI**	Data for 14/27 patients		Data for 15/28 patients
MRI abnormalities			
Basal ganglia^2^ (age at diagnosis)	14% (2/14) (median age 5.5 Y)		13% (2/15)
Lateral ventricles^3^ (age at diagnosis)	21% (3/14) (median age 5 Y)		20% (3/15)
Brain atrophy (age at diagnosis)	21% (3/14) (median age 7 m)		20% (3/15)
Delayed myelination (age at diagnosis)	7% (1/14) (4 m)		7% (1/15)
No abnormalities (age at MRI)	50% (7/14) (age not supplied)	5 m – Neuroimage normal	53% (8/15)
**Epilepsy**	100% (27/27)	+	100% (28/28)
Age at diagnosis (median, IQR)	Median 3.75 m (Onset 0–18 m:2 m)	4 m	Median 4 m
Initial phenotype			
IS (age at diagnosis)	85% (23/27) (median 4, 1.95; m)	+	85% (24/28)
Ohtahara (age at diagnosis)	4% (1/27)		4% (1/28)
Other – tonic clonic (age at diagnosis)	15% (4/27) (median 4.5, 8; m)		14% (4/28)
Later phenotype(s)		No ongoing seizures at 2.5Y	
Focal seizures	7% (2/27)		7% (2/28)
Myoclonic jerks (age at diagnosis)	15% (4/27) (median 2.5, 3.25 Y)		14% (4/28)
Tonic spasms, tonic clonic, and frontal lobe epilepsy (age at diagnosis)	22% (6/27) (median 4, 3.3 Y)		21% (6/28)
Treatments reported to be of benefit	ACTH, vigabatrin, phenytonin, phentobarbitol, felbamate, zonisamide, sodium valporate	Topiramate	
**Movement disorders**	93% (25/27)*	+	93% (26/28)
Age at diagnosis (median, IQR)	15/27 reported age of onset 2 to 11 months (5 m, 1.5 m)	7 m	
Type (age at diagnosis often not reported)			
Dystonia	52% (13/25)	+ (report details in the text)	53% (14/26)
Chorea	28% (7/25)	+	30% (8/26)
Dysphagia	4% (1/25)		4% (1/26)
Episodes of status dystonicus	12% (3/25)	+	15% (4/26)
Abnormalities of tone	64% (16/25)		61% (16/26)
Hypotonia	88% (14/16)	+	88% (15/17)
Spasticity	43% (7/16)	−	41% 7/17)
**Family history**		Maternally inherited	

2 Basal ganglia: cavitated, fragmented, indistinct, small, or normal (Kato et al, 2004).

3 Lateral ventricles: mildly to moderately enlarged lateral ventricles, sometimes in continuity with an interhemispheric fluid space (Kato et al, 2004).

* Remaining two patients are deceased and complete clinical assessments were not undertaken or reported.

# 8 *de novo* cases.

Variants in Families B, C, D, E, and F are classified as variants of uncertain clinical significance (VUS). There are reports of triplet expansions increasing the pA1 tract by one, two, and three residues (Bienvenu et al. 2002; Gronskov et al. 2004; Oegema et al. 2012). The pathogenicity of these shorter expansions is not well established. In this study, there are four cases of diminishing size of duplicating/insertion GGC triplet repeats, with a novel four GCG triplet repeat: c.306GGC[14], leading to a pA1 tract of 20 alanines, one case of a three GGC triplet repeat: c.306GGC[13], and two cases of a single addition of a GCG triplet repeat: c.306GGC[10];[11] and: c.306GGC[11]. The last case describes a 12 bp deletion: c.306GGC[6] leading to a shorter pA1 tract of 12 alanines.

### Polyalanine tract 2 variants

The most prevalent *ARX* mutation, a 24 bp duplication in exon 2 leading to expansion of pA2 to 20 alanines, c.441_464dup (herein named as dup24), was identified in eight patients, including one female, belonging to six distinct families (Families G to L) (Table S2). Interestingly, in the two brothers of Family H a recognizable episodic dystonic movement of the hands was identified in a retrospective revaluation (at 16 and 11 years old) allowing the diagnosis of the Partington-like syndrome (Fig. S3). Variants that are unlikely to be, or are certainly not pathogenic were identified in the remaining families with a pA2 variant (Families M to Q) (as shown in Table3). In cases of families M to P the same nucleotide region within pA2 was deleted. This deletion is classified as a variant that is unlikely to negatively influence ARX function. In Family Q we report a novel pA2 deletion of 15 base pairs, c.441_455del;[=] resulting in a contraction of five alanines in tract pA2 of ARX. Segregation studies in this family revealed that two healthy male relatives inherit the same variant. Analyses of 200 control chromosomes of healthy Portuguese controls did not detect this novel variant indicating that this contraction represents a very rare, benign polymorphism, and is not pathogenic.

## Discussion

Molecular analysis of *ARX* identified sequence variants in 17 families. There were seven families harboring disease-causing mutations, four families had unlikely pathogenic variants and in the remaining five families a variant of unclear/unclassified pathogenic significance (VUS) was identified, including one novel variant. Expansions of the first two polyalanine tracts in *ARX* account for more than half (56%) of all mutations previously reported in this gene (Shoubridge et al. 2010). Our strategy in the main (127/138 patients) was based on screening exon 2 by size-variant analysis (PCR and gel electrophoresis or multiplex analysis) followed by sequence analysis where indicated. Thirty of the 138 patients underwent sequencing of the entire *ARX* coding regions, many after a negative outcome by size-variant analysis. Of the 8 variants identified, 5 *ARX* variants were detected using multiplex screening that enables the simultaneous investigation of *FMR1*, *AFF2,* and *ARX* genes (Jorge et al. 2013) and three *ARX* variants were detected by direct sequencing of the gene. Despite these different approaches, all *ARX* variants identified in this study were located in exon 2 of the gene, specifically in pA1 and pA2. In our previous study, of the eight pathogenic variants identified only three were outside the pA tracts in a cohort of 613 mostly isolated cases, 500 of which had the entire open reading frame sequenced (Fullston et al. 2011). Hence, we predict the likelihood of missing a mutation elsewhere in the gene in the patients screened only by size-variant analysis would be quite low. From 138 patients we identified 7 known pathogenic variants, an overall detection rate of 5%. It remains challenging to assess the true prevalence of *ARX* mutations given the potential ascertainment bias. Here, we report a small number of mostly isolated cases and some brother pairs with a rate higher than the 1.3–1.5% from isolated cases (de Brouwer et al. 2007; Fullston et al. 2011), and 2.2% from brother pairs (Poirier et al. 2006) but substantially less than the 9.5% reported when screening large XLID families (Poirier et al. 2006).

**Figure 1:**
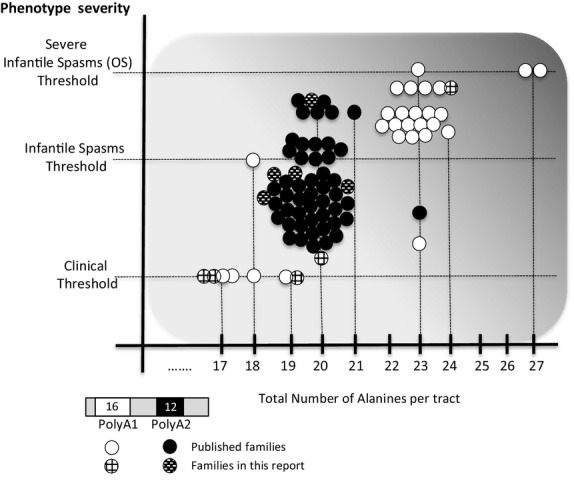
Clinical variability associated with expanded in polyalanine tract mutations in *ARX*. The panel below the graph shows the normal length of the pA1 as 16 residues and pA2 as 12 residues. The graph shows the relationship between the number of alanines and the phenotype severity in published families with pA1 (white circles) and pA2 families (black circles). Each circle represents a separate published case; some cases being a single affected individual whereas other cases are comprised of multiple affected individuals within a family. The black circle with horizontal line indicates a polyalanine tract expansion interspersed with a glycine residue (10A-G-12A) (Demos et al. 2009). The cases we are reporting in this study are shown as hatched circles. The same mutation can lead to different clinical outcomes, whereas different mutations can lead to consistent outcomes. With increasing length of residues in the pA tracts, the clinical presentation becomes more severe, with early onset seizures being a frequent but not consistent finding. Modified with permission from (Shoubridge et al. 2014).

*ARX* polyalanine tract expansion mutations of the same size and composition can cause a range of overlapping, but distinct clinical phenotypes (Fig.1). Each reported case is depicted as a single circle on the graph against the length of the tract involved. Some circles represent a single individual, whereas others represent multiple affected individuals within a family. For familial cases, the prevailing phenotype reported was used to designate the position on the axis of Figure1. This unusual breadth of variation in the clinical presentations, intra and interfamilial variability, associated with the frequent expansion mutations in pA1 and pA2 in particular has been noticed, but not adequately explained (Turner et al. 2002; Shoubridge et al. 2010). The clinical features of each of these cases are summarized in Table4 and Table S2, respectively.

The most frequent mutation in pA1 expands the 16 reside tract to 23 alanines. To date there are 18 families reported (across 27 individuals), increasing to 19 families (and 28 individuals) with our report of Family A. This particular mutation is associated with severe clinical outcomes ranging from generalized dystonia in the absence of infantile spasms (Shinozaki et al. 2008), to the more common X-linked infantile spasms (Stromme et al. 2002a; Wohlrab et al. 2005; Guerrini et al. 2007; Poirier et al. 2008; Cossee et al. 2011; Mirzaa et al. 2013), through to infantile epileptic-dyskinetic encephalopathy (Reish et al. 2009) and Ohtahara syndrome (Demos et al. 2009) (Table4). The age of onset and severity of the phenotype also varies; with reports of rapid neurodegeneration and death within the first year of life (Absoud et al. 2009). An added complexity is the overlapping clinical spectrum with mutations in other regions of *ARX*, with a number of patients reported with the severe early infantile encephalopathy of Ohtahara syndrome due to mutations outside of expansions to pA1 and pA2. These mutations include early truncating mutations (suggested to re-initiate translation at a subsequent methionine) (Fullston et al. 2009) and missense and nonsense mutations in the *Aristaless* domain (Giordano et al. 2010; Kato et al. 2010; Eksioglu et al. 2011; Sartori et al. 2011; Bettella et al. 2012). This highlights the need for detailed case reports of patients with expanded polyalanine tract mutations (and other mutations) in *ARX* to better identify the key clinical features associated with these infantile spasms.

The most frequent pA2 mutation expands the 12 reside tract to 20 alanines. To date there are 46 cases/families reported with this mutation, increasing to 52 cases with our report of Families G to L. Clinical outcomes always include mild to moderate ID either as the only consistent clinical feature, or in association with dystonia, particularly of the hands known as Partington syndrome, though to infantile spasms or West syndrome (Shoubridge et al. 2014). No syndromic features were recognized at the first referral in 13 of 17 patients, identified with *ARX* mutations. A retrospective detailed evaluation of some cases with the dup24 mutation revealed features of an “*ARX*-syndrome.” Cognitive profile evaluation in three patients with ID, and subsequent clinical assessment of a maternal male relative also with ID, enabled identification of hand dystonia in each male patient. Thus, identification of the dup24 pathogenic mutation was essential for the diagnosis of Partington syndrome and for appropriate genetic counseling.

In addition to the well-established pathogenic expansions to both tracts there are several examples of triplet expansions increasing the pA1 tract by one, two, and three residues (Bienvenu et al. 2002; Gronskov et al. 2004; Oegema et al. 2012). The pathogenicity of these shorter expansions is not well established. In this study, we identified triplet repeat expansion increasing pA1 by one (Family D and E), three (Family C), and a novel 4 residues (Family B). In this last case, the novel mutation in pA1 expands the first tract to 20 alanines. This precise number of alanines is sufficient to cause pathogenicity when expanded in pA2. Hence, it is difficult to predict the pathogenic impact of this expansion in pA1. In the absence of functional studies to determine the in vivo pathogenic status, haplotype analysis, in-silico and population studies are of particular importance. When we examine the genotype–phenotype spectrum associated with all cases of expansions to pA1 and pA2 of *ARX* we see that our patients generally cluster quite well with previously reported patients with the same mutations (Fig.1). However, there are outliers that do not fit with the genotype–phenotype correlation of a more severe outcome with a longer length of expansion. The novel mutation increasing the pA1 from 16 to 20 alanines in Family B gives rise to a phenotype more severe than seen in many patients with pA2 mutations increasing pA2 to 20 alanines in length. Although the authors have listed this mutation as a variant of unknown significance, additional cases may add weight to the potential pathogenicity of this mutation. Differential diagnoses are being considered for this patient. In the case of Family D, the variant was reported in a female patient. The variant in pA1 was of unknown significance, with the alanine tract increasing from 16 to 17 alanines (Family D). The female proband in Family D was also diagnosed with classic galactosemia, which can explain a mild intellectual impairment even in patients who avoid galactose. There is one other report of this change (Gronskov et al. 2004). Pathogenicity should not be ruled out in the variant c.331_333dupGCG, nor in others that cause a small increase in alanine residues of pA1 with further evidence needed to clarify the pathogenicity of small increases to alanine content in this tract.

There were three other cases in which females were identified with an *ARX* variant, this time in pA2 (Family L, P and Q). The female in Family L heterozygote for dup24 was lost to follow-up. The remaining two female cases had deletions in pA2. Deletions in pA2 are difficult to interpret in terms of pathogenicity (Shoubridge et al. 2010). The heterozygous c. 441_464del in one case from our cohort (Family P) was considered as a causative mutation in a male patient with nonsyndromic mental retardation previously published (Troester et al. 2007). Troester et al. interpreted the heterozygous deletion in the patient's asymptomatic mother and sister as a result of favorable X-inactivation in the brain. Evidence from several other publications places in frame deletions in exon 2, leading to retraction of pA1 and pA2, in an unlikely pathogenic group, based in part on some of these deletions also being reported in healthy, unaffected males (Bienvenu et al. 2002; Gronskov et al. 2004; Conti et al. 2010; Fullston et al. 2011). In agreement, the c.44a_455del;[=] variant identified in the female proband of Family Q was paternally inherited and did not segregate with cognitive impairment in the family, suggesting a nonpathogenic variant classification. The last case identified a previously unreported, presumed rare polymorphism (HGVS: c.441_455del; [=]) of paternal origin was identified in a heterozygous state in one family (Family Q).

Due to the overlap of phenotypic features in *ARX*-related and Fragile-X syndromes and family history of X-linked inheritance pattern, patients previously classified as having a normal *FMR1* profile by Southern blot irrespective of their phenotypic presentation were subsequently screened for an *ARX*-related disorder (exon 2 size-variant). Clinical details given by the referring physicians requesting screening for fragile-X syndrome often only relates specifically to fragile-X diagnosis. Consequently, in many cases there is little or no available clinical information specifically referable to *ARX*-related phenotypes. Given the outcomes of our screening strategy, we recommend that all patients referred for fragile-X (after confirming a normal karyotype), particularly those with a priori nonsyndromic ID or XLID, but also including cases with isolated cognitive impairment, be routinely screened for exon 2 size variants in *ARX*.

This study aims to establish standard nomenclature for *ARX* sequence variation description based on the published guidelines of the HGVS. One of the pitfalls in standardization of description of genetic variants concerns the absence of electropherogram visualization, hindering an effective variant comparison. However, variable description of *ARX* variants, combined with the low prevalence of familial cases and in many cases a lack of functional studies, hampers the establishment of pathogenicity and leads to confusion and misinterpretation, particularly in medical diagnostic settings. Hence, we have collated and compared the nomenclature of the variants to clarify this process and provide a useful resource. We encourage clinical researchers and clinical laboratory specialists to use Locus Specific Databases (LSDBs) (such as LOVD for *ARX* gene) to continue the process of collecting and describing identified sequence variants (http://databases.lovd.nl/shared/variants/ARX). This can ultimately be utilized to suggest and discuss accurate variant terminology and ensure clarity of variant descriptions essential for proper genetic counseling.

## Conclusion

The screening of mutations in the exon 2 of the *ARX* gene, followed by sequencing of the entire *ARX* gene, enabled the diagnosis of *ARX*-related disorders in eight patients from six families, presenting with different clinical phenotypes. Mutations were described using standardized and consistent nomenclature. In addition, several other pA1 expansions including the novel mutation expanding the first pA tract by four alanines in one family remain of unknown clinical significance. Further studies are required to characterize the impact of these variants on the functionality of ARX. Molecular screening of *ARX* gene is frequent in patients with XLID, ID presenting with infantile epilepsy or movement disorder or brain and genital malformations. Data from this and other studies led us to recommend that molecular screening of exon 2 of the *ARX* gene be done in all patients referred for fragile-X (after a normal karyotype), particularly those with apparent nonsyndromic ID of unknown cause, or in which the “Partington” hand dystonia is recognized.

## Supporting Information

**Data S1.** Supplementary note. **Figure S1.***ARX* mutations identified. **Figure S2**. Differences in regions designated as duplicated in pA2 expansion mutations of *ARX*. The sequence spanning c.421 and c.471 is shown encompassing pA2 of ARX. The region duplicated is indicated for the existing published nomenclature (underlined and in bold) as compared to the HGVS nomenclature (dotted box) for the 24dup (top panel), 27 bp (middle panel), and the 33 bp duplication (bottom panel). **Figure S3.** Block design subtests of WISC-III (Wechsler Scale), performed by the younger brother from Family H, demonstrate his inability to make purposeful hand movements due to lack of manipulative skills, thus suggesting hand dystonia. **Table S1.**PCR primers designed to amplify pA1 and pA2 or *ARX* ORFs. **Table S2.** Clinical features of patients with an *ARX* mutation leading to expansion of polyalanine tract 2 to 20 alanines (dup 24).
