# Supplementary material for: Unraveling the pathogenesis of ARX polyalanine tract variants using a clinical and molecular interfacing approach
Source: Mol Genet Genomic Med. 2015 Feb 25;3(3):203–14. doi: 10.1002/mgg3.133 (PMC4444162; doi:10.1002/mgg3.133)
Supplement: Supplementary file 1 — Data S1. Supplementary note. Figure S1.ARX mutations identified. Figure S2. Differences in regions designated as duplicated in pA2 expansion mutations of ARX. The sequence spanning c.421 and c.471 is shown encompassing pA2 of ARX. The region duplicated is indicated for the existing published nomenclature (underlined and in bold) as compared to the HGVS nomenclature (dotted box) for the 24dup (top panel), 27 bp (middle panel), and the 33 bp duplication (bottom panel). Figure S3. Block design subtests of WISC-III (Wechsler Scale), performed by the younger brother from Family H, demonstrate his inability to make purposeful hand movements due to lack of manipulative skills, thus suggesting hand dystonia. Table S1.PCR primers designed to amplify pA1 and pA2 or ARX ORFs. Table S2. Clinical features of patients with an ARX mutation leading to expansion of polyalanine tract 2 to 20 alanines (dup 24). [file mgg30003-0203-sd1.doc]

**SUPPLEMENTARY NOTE**

***Families: Description and screening outcomes***

*Family A.* The index case was born at term, after an uncomplicated pregnancy and delivery, subsequent to two previous miscarriages. Developmental delay was first noted at eight weeks of age, although the patient’s parents reported smiling and visual attention prior to this time. A plateau in development was noted from 8 weeks until 3 to 4 months of age, followed by developmental regression. At age 2.5 years he has severe to profound global developmental delay. He is able to roll, attempts to reach objects, makes some noises, smiles responsively and can briefly fix and follow objects visually. Infantile spasms began at four months of age, and an EEG at this time showed modified hypsarrhythmia with no focality. Spasms were refractory to high dose prednisolone, vigabatrin, pyridoxine and folinic acid. A tonic seizure was captured on repeat EEG at six months of age (while spasms were ongoing), but this seizure type was not reported or recorded at other times. Spasms ceased following introduction of topiramate at six months and did not recur. EEGs following resolution of the spasms showed a slow background with lack of expected sleep architecture but no epileptiform abnormalities at 7 months, and no significant abnormality at 15 months. At 2.5 years of age the patient has no ongoing seizures and is not on any anticonvulsants. A movement disorder with dystonia and choreoathetosis was noted from four months of age, with episodes of dysconjugate eye movements seen prior to onset of infantile spasms. However, the movement disorder was not prominent until the first dystonic crisis at seven months. Dystonic crises often occurred in the setting of recurrent illness, and resulted in respiratory distress, inability to feed due to the movements, and mild elevation of creatine kinase. The movement disorder required a number of hospitalisations, and was most problematic between 18 and 24 months of age. Treatment with clonazepam and baclofen provided some benefit, and is ongoing. At age 2.5 years, the movement disorder is still present, and remains severe at times, with chorea more prominent than dystonia, and sequelae including irritability, disturbed sleep and interference with motor function. Other clinical features include axial hypotonia, exotropia and plagiocephaly. Growth parameters are normal, and the patient is able to feed orally. MRI at 5 months of age was reported as normal. Levels of CSF neurotransmitters HVA and biopterins were borderline low.

A mutation in the *ARX* gene was suspected. Direct sequencing of PCR products from patient DNA demonstrated a duplicating/insertion of seven tandem GCG repeats within the normal stretch of 10 GCG triplet repeats within exon 2. This mutation results in the expansion of the first polyalanine tract (100 to 115 amino acids) of ARX from 16 to 23 residues. Sequencing of exon 2 in the mother confirmed heterozygote carrier status for this mutation.

*Family B*

A twenty month old boy was referred to for genetics consultation with congenital microcephaly, psychomotor delay, spasticity and dystonia. Examination showed hypotonia, hyperreflexia and nystagmus, with a micropenis (hypoplastic genitalia) and cryptorchidism Brain MRI, at 2 months of age, showed corpus callosum agenesis and simplified gyral pattern, more pronounced in the fronto-pariental regions. Epilepsy was diagnosed at 5 months, and responded to treatment with anticonvulsivants. EEG showed slow activity in the parietal, temporal and occipital regions and paroxysmal activity in the parietal and temporal regions. There was severe motor and language delay. At four years of age the proband only stands with support and is non-verbal. He shows agitation and sleep disturbance. Diagnosis of X-linked lissencephaly with ambiguous genitalia [OMIM#300215] was suspected thus the entire *ARX* ORF of the index case was analyzed. A novel hemizygous duplicating/insertion of unknown significance of four repeats of GCG resulting in an expansion from 16 to 20 alanines of pA1 was identified. His mother, who is healthy and has normal intelligence, is heterozygous for the same variant and has random X-inactivation (HUMARA assay)

*Family C*

A four year old boy was referred for motor, cognitive and speech delay. At birth, his weight was 2.7 kg (<3^rd^ percentile), length was 48 cm (<3^rd^ percentile) and head circumference was 34 cm (10-25^th^ percentile). Motor and language developmental milestones were delayed. He sat at 11 months, walked at 17 months and used a few single words by the age of 26 months. Brain MRI performed at two years and then repeated at age six, was normal with the exception of lateral ventriculomegaly observed only on the initial examination (age 2). Developmental evaluation at age of six years and four months identified impaired cognitive and emotional skills and a moderate attention deficit, which necessitated treatment with methylphenidate. The proband underwent occupational and speech therapy. Craniofacial examination, performed at 13 years of age, showed prominent ears and supraorbital ridges, large eyes, and overlapping toes.

The previously published hemizygous duplicating/insertion of unknown significance of three copies repeats of GCG resulting in an expansion from 16 to 19 alanines in the pA1 of the ARX protein, was identified after *ARX* exon 2 sequencing following multiplex screening. He was classified as having a global non-syndromic X-linked intellectual disability phenotype [OMIM#300419].

*Family D*

A four year old girl was referred with developmental delay and a behaviour disorder. At eight days of age the presence of jaundice, food intolerance, enlarged liver, hepatic dysfunction and failure to thrive lead to clinical suspicion of classic galactosemia. The diagnosis was confirmed by enzymatic and genetic study. After a free-lactose diet, she was asymptomatic.

Psychomotor development was normal in the first two years of life, as were her liver function, blood glucose and enzymatic assays. Developmental delay was noted by the age of two, with irritability and behaviour consistent with autism spectrum disorder which did not respond to risperidone treatment. There was subsequent regression in cognitive development in the absence of recognized seizures.

Physical examination at age of three years revealed no dysmorphic features, normal eye examination and normal hearing confirmed by formal audiology. Brain MRI identified delayed myelination. The molecular study for fragile-X revealed that she was not a carrier of the full mutation. Subsequent analysis of *ARX* identified a heterozygous variant of 3 GCG repeats.

*Family E*

The proband is a ten-year-old boy, referred for mild ID, hyperactivity and attention deficit. The proband’s older brother has learning disabilities and expressive language impairment, and the younger brother shows a similar language deficit. Early psychomotor development was normal although he received speech therapy from age two years. He was unable to read at seven years and three months of age and behavioural problems were reported. Physical examination showed no dysmorphic features. Array CGH was normal. The previously published and still uncharacterized hemizygous duplicating/insertion of a single GCG insertion resulting in an expansion from 16 to 17 alanines in the pA1 of the ARX protein, was identified in the proband after *ARX* exon 2 sequencing following the multiplex screening. This family was referred for a genetics consultation, but as yet no other family member has been counselled.

*Family F*

A ten year old boy was referred for genetics consultation for ID. No family history was available. He had myopia, deafness and scoliosis due to the presence of two lumbar hemivertebrae. Developmental examination showed cognitive delay but this has never been formally evaluated. Growth parameters at first observation were 5^th^, <5^th^ and ≤2^nd^ percentile for weight, height and OFC, respectively. Physical examination revealed a high forehead, triangular face, narrow palpebral fissures, prominent nasal root, large mouth with thin upper lip, protruding jaw, short thorax due to scoliosis. CT head was normal with the exception of presence of circumscribed areas of osteolysis in the parietal bone. Sequencing of *ARX* exon 2, following the multiplex screening, revealed a hemizygous 12bp deletion in pA1, affecting alanine number, which is reduced by four units.

*Family G*

The three and a half year old male proband presented at six weeks with stiffening episodes lasting two to three minutes. These persisted for two to three weeks and then ceased. At seven months he had unusual posturing with back arching and increased tone. By 11 months he was seen by a paediatric neurologist and was diagnosed with infantile spasms. EEG showed hypsarrythmia and brain MRI was normal. Array CGH revealed a 340 kb duplication at Xp22.31, containing the pseudoautosomal region of the short arm of the sex chromosomes (PAR1; X and Y chromosomes cannot be distinguished at this region due to sequence homology). There are no genes in the duplicated region on the X chromosome, present also in the mother, and thought to be a variant of unknown significance. Seizure control was achieved by 16 months with valproate therapy.

Developmental delay was evident by age six months although he sat at nine months. Unassisted walking did not occur until 20 months. He has not developed a pincer grip to date. At 27 months he has no words. He has some role play and is able to wave good-bye and clap. On examination, he has minor dysmorphic features, including an open-mouth appearance with drooling, ptosis with hooded eyes, foetal fingertip pads with 5^th^ finger clinodactyly and bilateral 2 - 3 toe syndactyly. He is mildly hypotonic with large joint hyperflexibility.

Molecular screening of exon 2 of *ARX* identified the dup24 mutation in pA2. Sequencing of exon 2 in the mother identified her to be a heterozygote carrier for this mutation.

*Family H*

Two brothers were referred at two and seven years of age, due to ID and congenital macrocephaly (the latter excluded in both parents). Both had normal growth weight and length at birth. Attention deficit hyperactivity disorder was noticed in the first years of life. The oldest brother had two febrile seizures at the age of 2.5 years and showed facial asymmetry. Brain MRI was normal at the age of three. The youngest brother has minor facial dysmorphic features, and had one episode of rolling of the eyes, with normal brain MRI and EEG. Sequencing of *ARX* exon 2 following the multiplex screening, allowed the characterization of the dup24 in pA2 in the brothers and confirmation of heterozygote carrier status for this mutation in their mother.

*Family I*

A four year old male proband, was referred due to ID and attention deficit. He had growth retardation with prenatal onset, presenting an unusual, slightly dysmorphic face with prominent ears. He attended a speech and language therapist. He had a maternal uncle with ID. Sequencing of *ARX* exon 2 after the multiplex screening, allowed the characterization of a hemizygous dup24 in the proband and his maternal uncle, as well as the heterozygote carrier status in multiple generations of female carriers. The proband’s neuropsychological evaluation revealed mild global developmental delay, as well as specific difficulties on tasks requiring manipulative and fine motor skills and manual dexterity. The proband’s maternal uncle also showed hand “reach and grip” impairment, allowing the diagnosis of Partington-like syndrome in this family.

*Family J*

An eight year old boy, was referred with ID. He walked at 18 months and at the age of three years started speech development. Since the age of three years, sleep disturbance, attention deficit, impulsivity, auto-aggression and obsessions were noticed. Seizures as well as developmental regression were not reported. He had non-specific craniofacial dysmorphism. Sequencing of *ARX* exon 2 following the multiplex screening, allowed the characterization of the dup24, that was absent in DNA obtained from his healthy mother blood sample, indicating a neo-mutation (*de novo* or gonadal mosaicism).

*Family K*

The five year old proband was referred due to motor impairment, severe language delay and hyperactivity. At nine months old he sat unattended, and at 17 months he was walking. By then, speech delay was noted as he used a few single words. At the age of four and a half, the proband’s growth parameters were all on the 95^th^ percentile. On physical examination, he had bilateral epicanthus, ataxia, joint laxity and hand “reach and grip” impairment and babbled in a conversational manner. Methylphenidate was used to treat ADHD with improvement in attention and impulsivity. Sequencing of *ARX* exon 2 following the multiplex screening, allowed the characterization of the dup24, and the heterozygote carrier status for this mutation in proband’s mother. His mother has random X-inactivation (HUMARA assay)

*Family L*

A ten year old girl was referred with ID. Her mother also had severe learning difficulties. Motor and language delay were noted. The proband walked at two years of age and was had very limited language by the age of three. Her school performance was very poor; she was unable to write, recognizing only a few words. On physical examination at the age of ten, she showed no dysmorphic features but had growth parameters above 90^th^ percentile, being clinically obese (BMI>95). Sequencing of *ARX* exon 2 after following the multiplex screening, allowed the characterization of the dup24 in a heterozygous state. X-inactivation studies (HUMARA assay) [46] detected no significant deviation.

*Family M*

A four-year-old boy referred for delayed psychomotor development. Developmental examination showed delayed fine motor skills, and receptive language impairment, with fluent speech and no dysmorphic features. The previously published hemizygous 24bp deletion, resulting in the contraction from 12 to 7 alanines in the pA2 of the ARX protein, was identified after *ARX* exon 2 sequencing following the multiplex screening.

*Family N*

A thirteen year old boy was referred for learning disability. At the age of five months he underwent surgery for a volvulus, . Language impairment was noticed by the age of 3 to 4 years. He had mild cognitive impairment and behavioural problems. Sequencing of *ARX* exon 2 following the multiplex screening, allowed the characterization of the previously published hemizygous 24bp deletion, resulting in the contraction from 12 to 7 alanines in the pA2 of the ARX protein.

*Family O*

An eight year old boy was referred for learning disability and attention deficit. Wechsler Scales (WISC-III) measures of developmental and intellectual performance were below normal range. From twelve months of age he had four febrile seizures. EEG studies were normal. He had delayed development, frequent crying and a difficult adaptation to kindergarten. Physical examination was normal, with no major dysmorphism. Karyotype showed a mosaic: mos 47, XYY (47)/46, XY(5). Additionally, sequencing of *ARX* exon 2 following the multiplex screening, allowed the characterization of a hemizygous 24bp deletion, with unknown significance, resulting in the loss of 8 alanines in the pA2 of the ARX protein. His mother is a heterozygous carrier for this variant.

*Family P*

An eleven year old girl was referred with ID. A maternal uncle with severe ID and seizures of unknown aetiology died at eighteen years of age. She had normal general health and has completed the 4^th^ year of elementary school with an adapted curriculum and support. She is shy, distracted, anxious and slow, shows memory impairment, but has good practical skills. Sequencing of *ARX* exon 2 following the multiplex screening, allowed the characterization of the previously published heterozygous 24bp deletion, resulting in the contraction from 12 to 4 alanines in the pA2 of the ARX protein. X-inactivation studies (HUMARA assay) [46] detected no significant deviation.

*Family Q*

A ten month old girl was referred for facial dysmorphism. During infancy there was mild to moderate cognitive impairment. Growth parameters at eight years of age were 25-50th, <2th and 10th percentile for weight, height and OFC, respectively. Examination showed a peculiar and flattened face, ptosis, broad nasal root, pinched bulbous nasal tip, protruding ears, bilateral epicanthus with down slanting, narrow and elongated palpebral fissures, down-turned corners of mouth, short neck and normal hearing. Ophthalmologic evaluation showed myopia and myopic astigmatism. She underwent a plastic surgery procedure to lower eyelid suspension by canthopexy/canthoplasty. Sequencing of *ARX* exon 2 following the multiplex screening, allowed the characterization of a novel 15bp deletion, resulting in the contraction from 12 to 7 alanines in the pA2 of the ARX protein. Several family members were studied, revealing paternal inheritance and also that this *ARX* variant did not segregate with cognitive impairment in this family.

**SUPPLEMENTARY DATA**

**Supplementary Table S1: PCR primers designed to amplify pA1 and pA2 or *ARX* ORFs.**

|  |  | |  |  |  |
| --- | --- | --- | --- | --- | --- |
|  |  | |  |  |  |
|  |  | | **Primer sequence 5' → 3'** | |  |
|  | **Region/exon** | | **Sense** | **Anti-sense** | **Expected size (bp)** |
| *Australian cohort* | **exon 1** | | gtccactacacttgttaccgc | aattgacaattccaggccactg | 520 |
|  | **exon2-pA1** | | ctgatagctctcccttgccc | gcggcccctgcgccgtccggccgttc | 262 |
|  | **exon2-pA2** | | cccctccgccgccaccgccaac | tcctcctcgtcgtcctcggtgccggt | 313 |
|  | **exon 2c** | | gcaagtcgtaccgcgagaacg | cagctcctccttgggtgaca | 371 |
|  | **exon 2d** | | aactgctggaggacgacgagg | tgcgctctctgccgctgcga | 392 |
|  | **exon 3** | | gaaatagctgagagggcattgc | tctcttggttttgtgaaggggat | 231 |
|  | **exon 4** | | gacgcgtccgaaaacaacctgag | ccccagcctctgtgtgtatg | 551 |
|  | **exon 5** | | acagctcccgaggccatgac | gagtggtgctgagtgaggtga | 347 |
| *Portuguese cohort* | **pA1 & pA2*** | | cagcagccctggctgggactc | HEX-cggtacgacttgctgcggctg | 380 |
|  | **exon 1** | | caacacacacccatccatccc | ccccgaacaccaaacatcc | 344 |
|  | **exon 2a** | | gcgtcgaagtctggtggtgc | gcctgggacacgctcaagat | 425 |
|  | **exon 2b** | | tcctgtggccgccactg | cccgctgtccctccctg | 454 |
|  | **exon 2c** | | gggacacgctcaagatcagc | ccgtccttgccctcagc | 345 |
|  | **exon 3** | | agaccctggtggagtaggcc | gccaccaacccatctctctc | 212 |
|  | **exon 4** | | tgggctctctctgccttgc | actcctgcctcctccctgc | 430 |
|  | **exon 5** | | ctcccgaggccatgacc | cttgagtggtgctgagtgagg | 346 |
|  | **M13 tail** | | tgtaaaacgacggccagt | caggaaacagctatgacc | - |
| * without M13 tail | | |  |  |  |
|  | |  |  |  |  |

CGGCAGCGGCGGCGGCGGCGGCGGCGGCGGCGGCGGCGGCA

**I.**


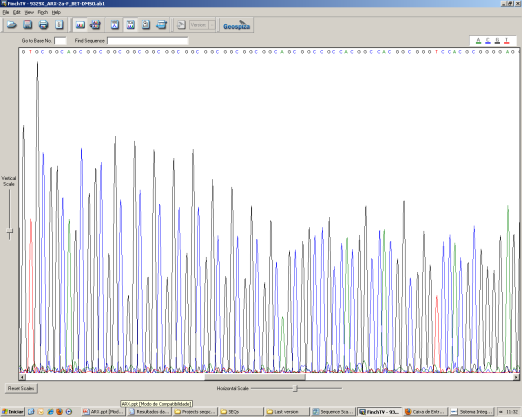


pA1


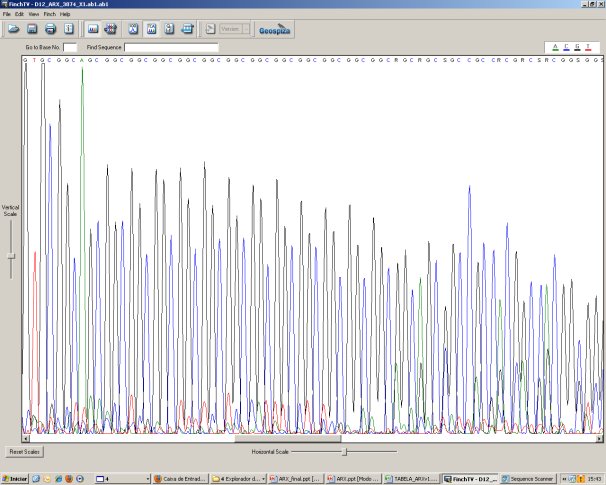

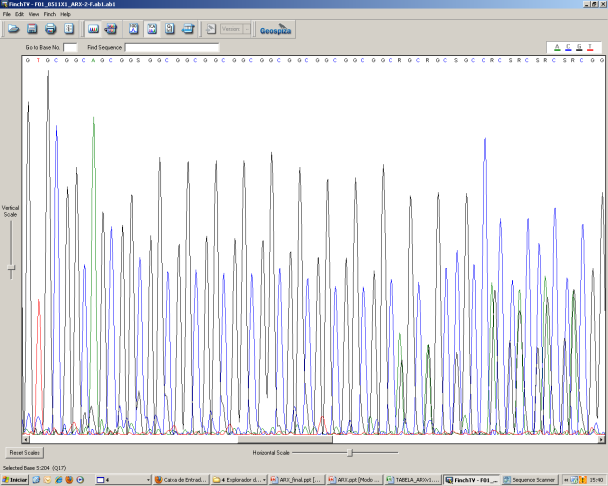


(a)

(b)

(c)

CGGCAGCGGCGGCGGCGGCGGCGGCGGCGGCGGCGGCGGCGGCGGCA


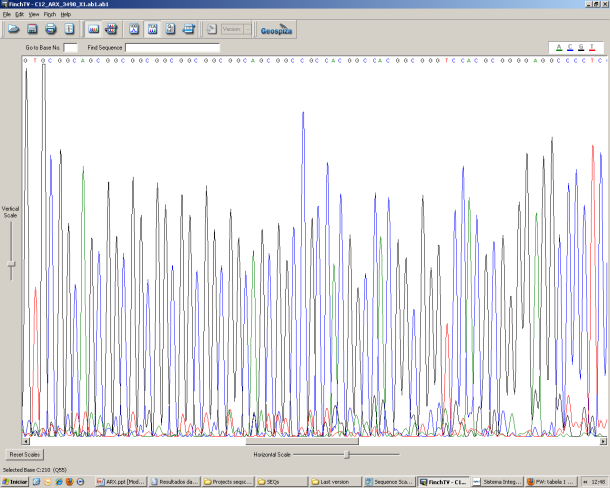


(e)


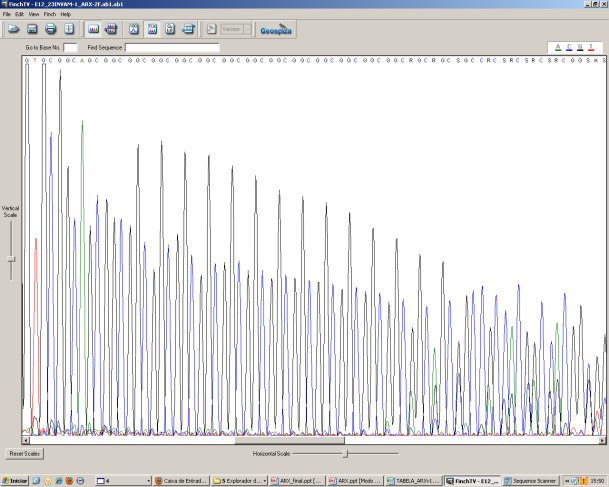


(d)

**I.**

CGGCAGCGGCGGCGGCGGCGGCGGCGGCGGCGGCGGCGGCGGCGGCGGCA

CGGCAGCGGCGGCGGCGGCGGCGGCAGCGGCCGCCA

GGCGGCGGCGGC

^


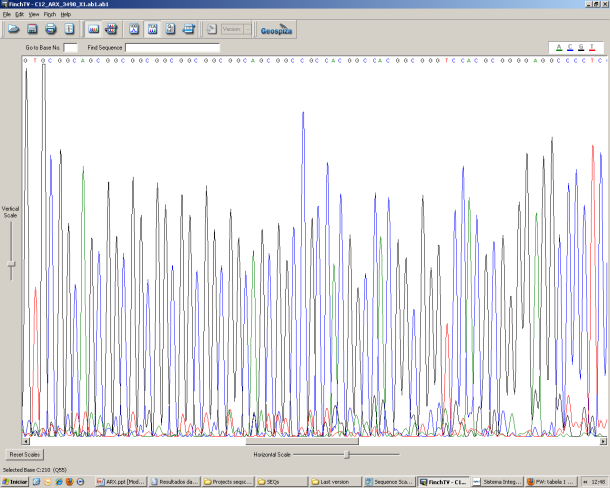


(e)


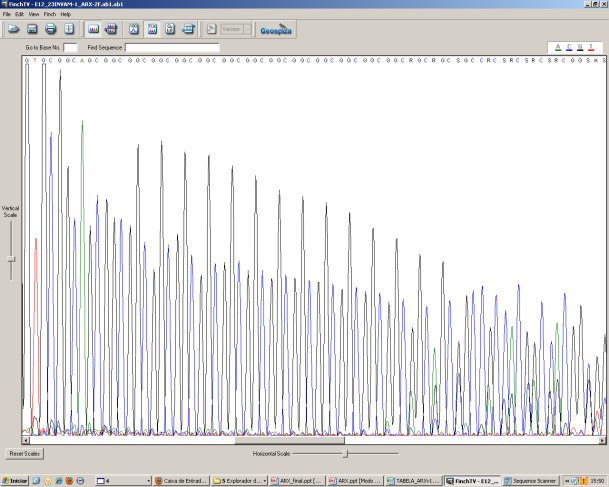


(d)

**I.**

CGGCAGCGGCGGCGGCGGCGGCGGCGGCGGCGGCGGCGGCGGCGGCGGCA

CGGCAGCGGCGGCGGCGGCGGCGGCAGCGGCCGCCA

GGCGGCGGCGGC

^


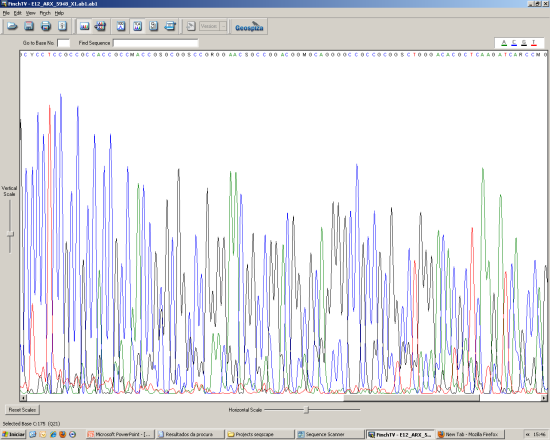


(d)

**II.**

ACGGCGCAGGGGCCGCCGCGGCCTGGGAC

Mut allele

Wt allele


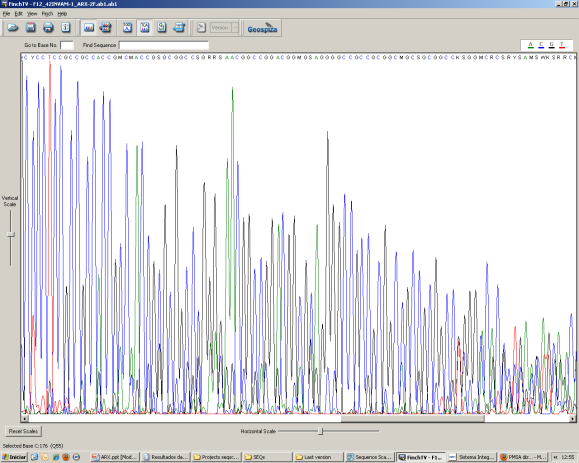


ACGGCGCAGGGGCCGCCGCGGCCGCCGCGGCCTGGG

^

ACGGCGCAGGGGCCGCCGCGGCAGCCGCGGCCGCGGCCGCCG

(e)

^

AGCCGCGGCCGCGGCCGCCGCGGC

**Supplementary figure F1 -** *ARX* mutations identified.

1. Partial electropherograms of the *ARX* exon 2 pA1 (sense strands). Normal sequence of pA1 (**a**). Mutations leading to expanded polyalanine tracts of *ARX*: c.306GGC[11] (Family D) (**b**); c.306GGC[13] (Family C) (**c**); c.306GGC[14] (Family B) (**d**); and a pA1 deletion c.306GGC[6] (Family F) (**e**).
2. Partial nucleotide sequence of the ARX exon 2 pA2(sense strand). Normal sequence of pA2 (**a**). Prevalent ARX mutation c.441_464dup (most frequently named c.429_452dup) in a male (Family K) (**b**) and a female (Family L) (**c**) patient. Deletions found in pA2: a male with the c. 441_464del (Family O) (**d**) and a female with the c. 441_455del (Family Q) (**e**).

G A G A A A A A A A A A A A A W D

GGC GCA GGG GCC GCC GCG GCA GCC GCG GCC GCG GCC GCC GCG GCC TGG GAC (24bp)

| |

c.421 c.471

G A G A A A A A A A A A A A A W D

GGC GCA GGG GCC GCC GCG GCA GCC GCG GCC GCG GCC GCC GCG GCC TGG GAC (27bp)

| |

c.421 c.471

G A G A A A A A A A A A A A A W D

GGC GCA GGG GCC GCC GCG GCA GCC GCG GCC GCG GCC GCC GCG GCC TGG GAC (33bp)

| |

c.421 c.471

**c.429_452dup** c.441_464dup

**c.430_456dup**

c.435_462dup

**c.423_455dup**

c.426_458dup

**Supplementary figure F2**. Differences in regions designated as duplicated in pA2 expansion mutations of *ARX*. The sequence spanning c.421 and c.471 is shown encompassing pA2 of ARX. The region duplicated is indicated for the existing published nomenclature (underlined and in bold) as compared to the HGVS nomenclature (dotted box) for the 24dup (top panel), 27bp (middle panel) and the 33bp duplication (bottom panel).

**
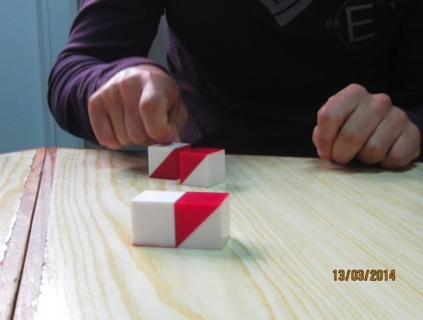
** **
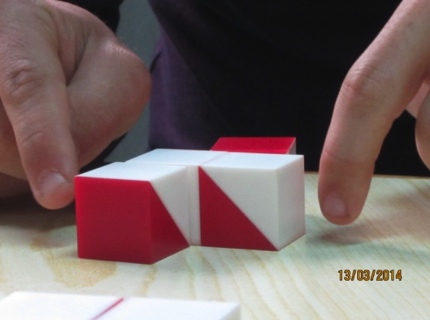
**

**Supplementary figure F3** – Block design subtests of WISC-III (Wechsler Scale), performed by the younger brother from Family H, demonstrate his inability to make purposeful hand movements due to lack of manipulative skills, thus suggesting hand dystonia.

**Supplementary Table S2:** Clinical features of patients with an *ARX* mutation leading to expansion of polyalanine tract 2 to 20 alanines (dup 24)

| **Family identification** | | **G** | **H** | | **I** | | **J** | **K** | | **L** |
| --- | --- | --- | --- | --- | --- | --- | --- | --- | --- | --- |
| **Family history** | | Brother with trisomy 21 (No *ARX* mutation) | Brother with ID | Brother with ID | Maternal uncle with NF1 | Sister with NF1 | - | Paternal cousin with FXS | | Mother with ID (unable to read) |
| **DD/ID** | |  | + | ++ | + | + | +/++ | + | | + |
|  | Age at diagnosis | 6M | 15Y | 10Y9M | 6Y | 39Y | 8Y6M | 4Y5M | | 10Y |
|  | Formal development assessment (IQ, age at evaluation) |  |  |  | GDQ 57 (5Y) | Not performed | Low IQ (8Y) | Motor impairment; language delay | | Unable to read |
|  | DD onset prior to seizure onset  (age at diagnosis) | 6M | 15Y | 10Y 9M | Unknown | No | Unknown | Unknown | | Unknown |
|  | Developmental regression |  | no | no | No | Unclear | No | No | |  |
| **Brain / behavioral disorders**  **(age at diagnosis)** | | None reported |  |  |  | No | Sleep difficulties  / agitation |  | | - |
|  | ADHD |  | + (first Y) | + (first Y) | + | No | + (3Y) | + | | - |
|  | Autistic behavior |  | - | - | + | No | - | + | | - |
|  | Impulsivity |  | - | - | + | No | + (3Y) | + | | - |
|  | Aggressive outbursts |  | - | - |  | No | Auto-agression (3Y) | - | | - |
|  | Other |  |  |  |  |  | Obsessions (3Y) |  | |  |
| **MRI assessment (age at evaluation)** | | Not reported | Normal (3Y) | Normal (2Y) | Normal | Not reported |  |  | | Normal CT |
| **Epilepsy (age at diagnosis)** | | ISS + hypsarrthmia (11M) | Febrile seizures (2Y) | Febrile seizures (? First Y) | Unknown | Isolated febrile seizure (~12M) | No seizures | No seizures | |  |
|  | Response to anticonvulsivants | Valporate (16M) |  |  |  |  |  |  | |  |
| **Movement disorders** | |  |  |  | + | + |  | Ataxia  (waddling gait) | |  |
|  | Dystonia (age at diagnosis) |  | + (16Y) | + | + | + |  | + | |  |
| **Hypotonic** | | Mild |  |  |  |  |  |  | |  |
| **Dysmorphisms / other** | | - | Macrocephaly | Macrocephaly | Prominent ears | + |  | Bilateral epicantus | | - |
| **Unspecific** | | Global speech delay |  | Broad forehead |  |  | Mild constipation | Joint laxity | | Obesity |
| **Global phenotype of the *ARX*-spectrum** | | *Partington-like syndrome* | | | | | | |  | *Non-syndromic XLID ?* |
